# Supplementary material for: A marine cryptochrome with an inverse photo-oligomerization mechanism
Source: Nat Commun. 2023 Oct 30;14:6918. doi: 10.1038/s41467-023-42708-2 (PMC10616196; doi:10.1038/s41467-023-42708-2)
Supplement: Supplementary file 1 — Supplementary Information [file 41467_2023_42708_MOESM1_ESM.pdf]

Supplementary Information for:

A marine cryptochrome with an inverse photo-oligomerization mechanism

Authors

Hong Ha Vu[‡1], Heide Behrmann[‡2], Maja Hanić[3], Gayathri Jeyasankar[2], Shruthi Krishnan[1], Dennis Dannecker[2], Constantin Hammer[1], Monika Gunkel[2]; Ilia A. Solov'yov[3,4,5], Eva Wolf*[1,6], Elmar Behrmann*[2]

‡ These authors contributed equally; * corresponding authors

Contents

Supplementary Figure 1

Supplementary Figure 2

Supplementary Figure 3

Supplementary Figure 4

Supplementary Figure 5

Supplementary Figure 6

Supplementary Figure 7

Supplementary Table 1

Supplementary Table 2

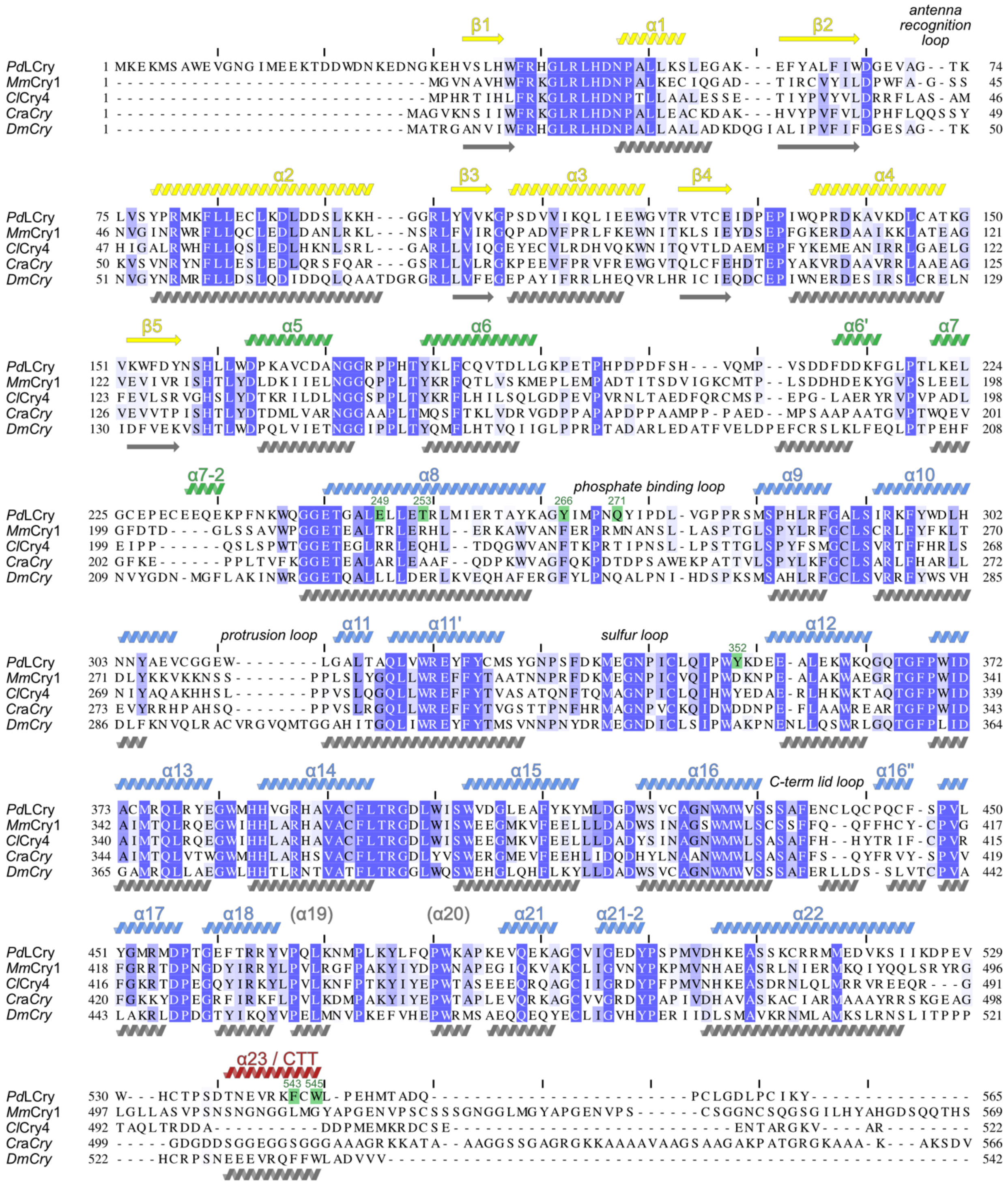

Supplementary Figure 1: Sequence alignment of animal- and animal-like CRYs with known structure.

Detailed sequence alignment of *Pd*LCry (NCBI AccNo. AGX93007), mouse cryptochrome 1 (*Mm*CRY1: P97784), pigeon cryptochrome 4 (*Cl*Cry4: ANT47196), green algae animal-like cryptochrome (*Cra*Cry: A8J8W0) and drosophila cryptochrome (*Dm*Cry: NP_732407). Secondary structure elements of *Pd*LCry (coloured according to the domain organisation of Fig. 1, based on our dark state structure) and *Dm*Cry (grey) are shown. For *Dm*Cry, these elements and their numbering are based on Czarna *et al.*[1] Amino acids mutated in this study are highlighted in green.

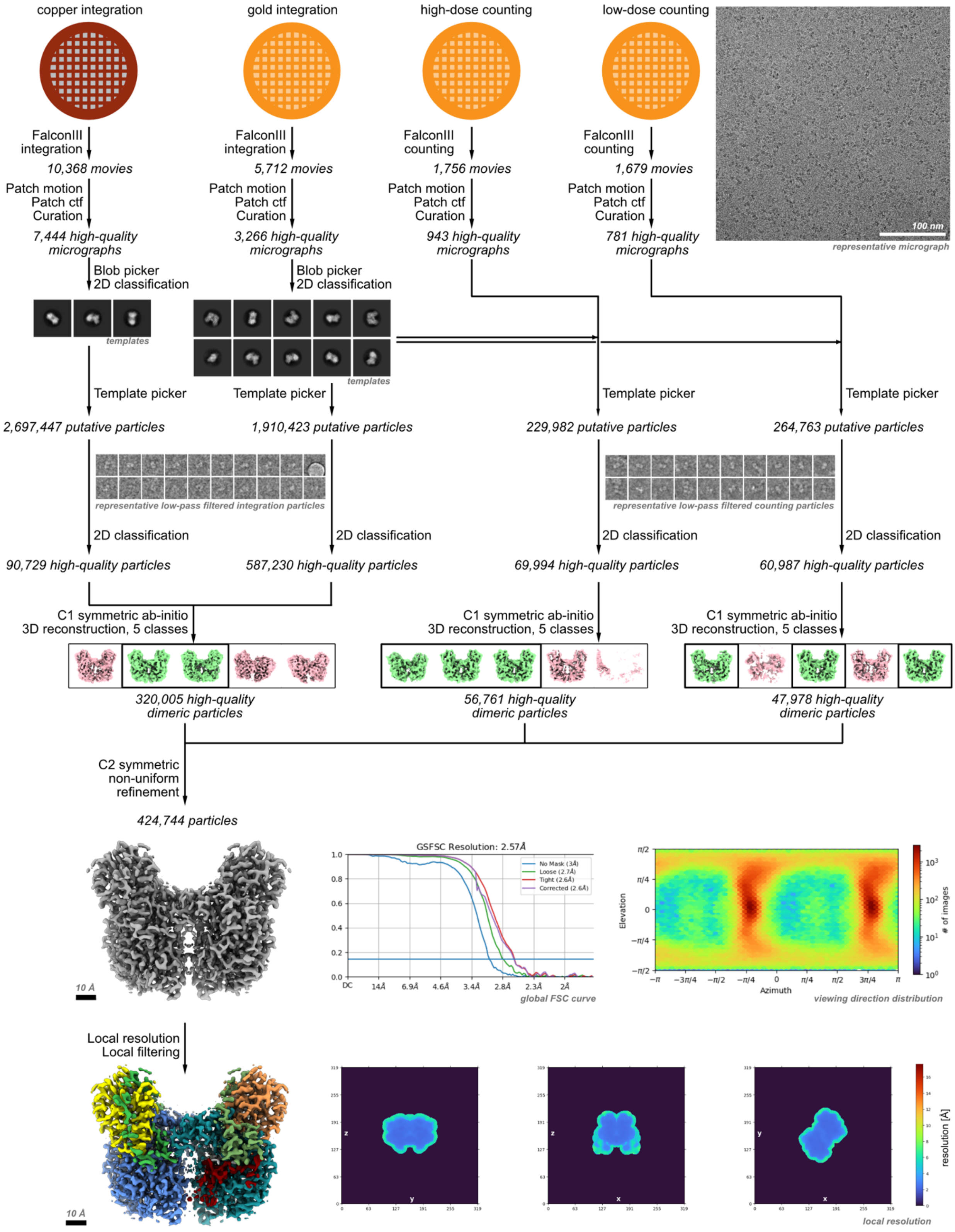

Supplementary Figure 2. Overview of the single particle cryo-EM data processing workflow for the dark state.

A total of 4 datasets were collected on a 300 kV cryo-electron microscope. Two of these datasets were acquired in the integration mode of the Falcon III detector, and the other two were acquired in the counting mode. For each dataset, movies were selected for low per-frame drift rates, good CTF scores, and low astigmatism. Particles were first picked using a blob picker, and then subjected to unsupervised 2D classification. This resulted, after rejecting clearly artificial classes, in a set of both monomeric (about 15%) and dimeric (about 80%) particles, with the remaining classes ambiguous regarding their oligomeric state. Representative classes were used for a template picker, and then curated using unsupervised 2D classification, selecting for particles with protein-like density and resolutions better than 7 Å.

The dimeric particles were further curated using ab-initio reconstruction, sorting them into 5 distinct populations. From these, all particles contributing to C2-symmetric dimeric structures (shown in green and highlighted by a thicker box outline) were combined and refined in 3D using a non-uniform refinement algorithm, resulting in a map with a uniform resolution of 2.6 Å. For further details, see the Materials and Methods, and Supplementary Table 2.

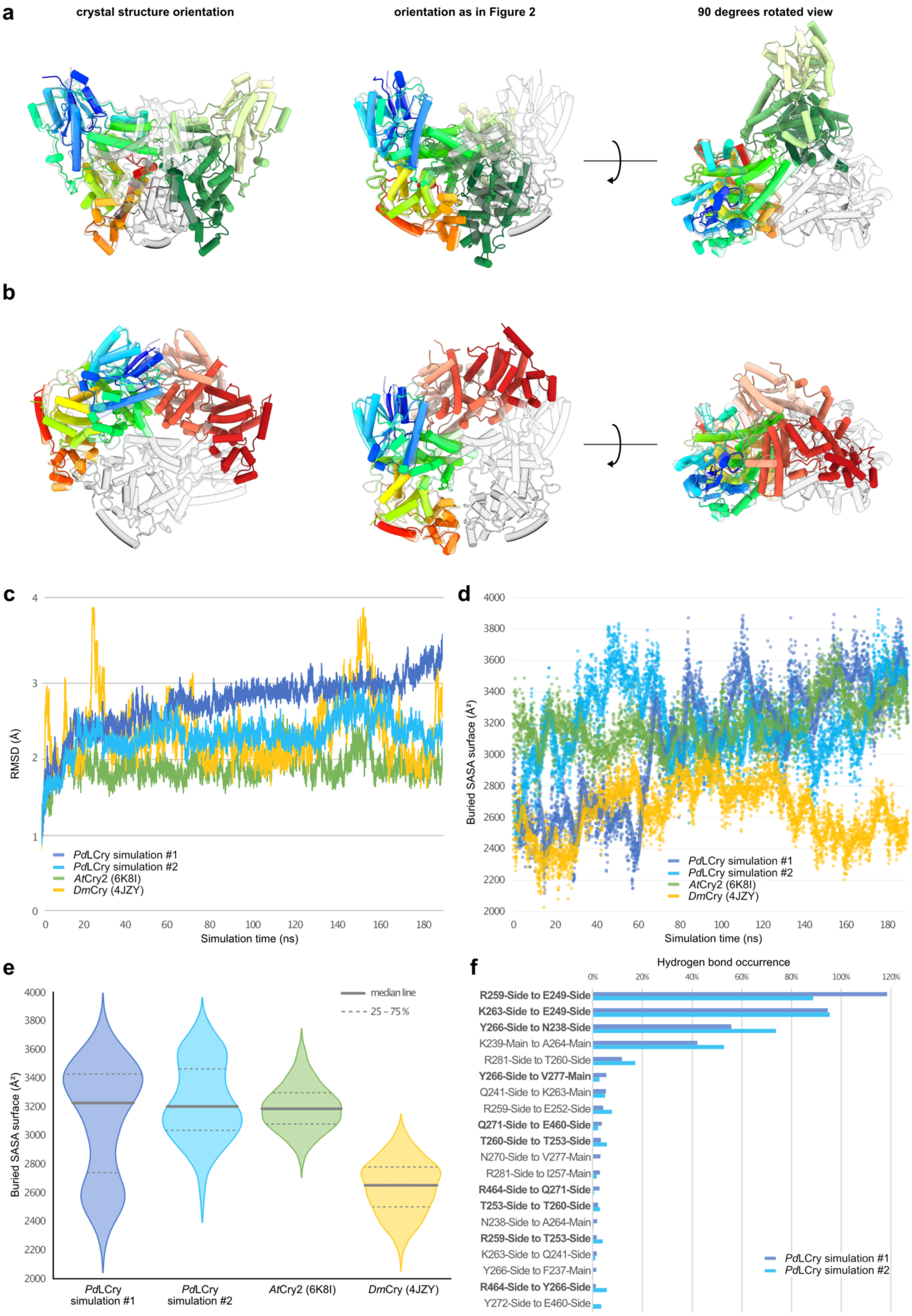

Supplementary Figure 3: Comparison of the *Pd*LCry dark state dimer with other CRY dimer arrangements.

a Comparison of the subunit arrangement in the *Dm*Cry crystal dimer and **b** in the dark state *At*Cry2 dimer with the arrangement in the dark state *Pd*LCry dimer (shown as transparent). For each, subunit 1 has been aligned to subunit 1 of the *Pd*LCry dimer. These subunits are depicted using a rainbow color gradient from blue (N-terminus) to red (C-terminus). Subunit 2 is depicted in a gradient from light green (N-terminus) to dark green (C-terminus) for *Dm*Cry (4JZY), from light red (N-terminus) to dark red (C-terminus) for *At*Cry2 (6K8I), and from transparent light gray (N-terminus) to transparent dark gray (C-terminus) for *Pd*LCry. **c** Evolution of the root mean square deviation (RMSD) of the dimeric backbone atoms from the reference configuration taken after the equilibrium simulations. The overall RMSD for the studied dimers converges to the values below 3 Å for the *At*Cry2 (green trace) and *Pd*LCry simulation #2 (light blue trace) which indicate stable dimers. RMSD for the crystal *Dm*Cry dimer simulation (orange trace) shows several peaks, which appear due to the slight relative oscillatory motion of the two monomers in the *Dm*Cry dimer structure. A slight increase of the RMSD in the case of the *Pd*LCry simulation #1 (dark blue trace) is also caused by relative motion of the two monomers, which remarkably was not seen in the replica *Pd*LCry simulation #2. This motion is reflected in the SASA time evolution shown in d, which demonstrates that in the case of *Pd*LCry simulation #1 the buried SASA value drops shortly after the beginning of the simulation, and then increases again to the characteristic value also seen in the case of *Pd*LCry simulation #2. Note that the RMSD peculiarities in the case of *Dm*Cry and *Pd*LCry simulation #2, however, are rather minor, as overall the RMSD stays well below 4 Å, indicating overall stable structures, as RMSD values for stable single proteins of the size of cryptochrome are expected to be within a 2-4 Å range[2–6]. **d** Evolution of the solvent accessible surface area (SASA) buried in the interaction interface during MD simulations of the *Pd*LCry dimer (two independent simulations, light and dark blue traces), the physiologically relevant *At*Cry2 dimer (green trace), and the crystal *Dm*Cry dimer (orange trace). Considering the first 190 ns of the simulation, *At*Cry2 dimers (SASA average 3194 ± 164 Å^2) and *Pd*LCry dimers (SASA average 3138 ± 343 Å^2) had on average larger buried interfaces compared to the unphysiological *Dm*Cry dimer (SASA average 2640 ± 189 Å^2). Individual values at each time point of the simulation are shown as transparent dots, the lines show the running average over 0.25 ns simulation time. **e** Violin plot representation of the calculated SASA values. The figure shows the mean values and indicates the corresponding 25-75% percentile intervals of the data. The skewed distribution for *Pd*LCry simulation #1 is due to the significantly lower SASA values at the beginning of the simulation compared to the results obtained after 190 ns. The average SASA values of the *Pd*LCry and the *At*Cry2 structures and their confidence intervals are well separated from the result obtained from the *Dm*Cry simulation. **f** Number of dimer-interface hydrogen bonds observed during the two independent *Pd*LCry MD simulations, with interactions involving residues that were studied in follow-up mutagenesis experiments (see Fig 6) shown in bold. The list is limited to hydrogen bonds involving residues of helix α8 and its flanking region (225 – 302), and of helix α18 (459 – 465), see Fig. 3. Since several sidechains can form more than one hydrogen bond simultaneously, occurrences above 100% are possible (e.g. Arg259 to Glu249 interaction).

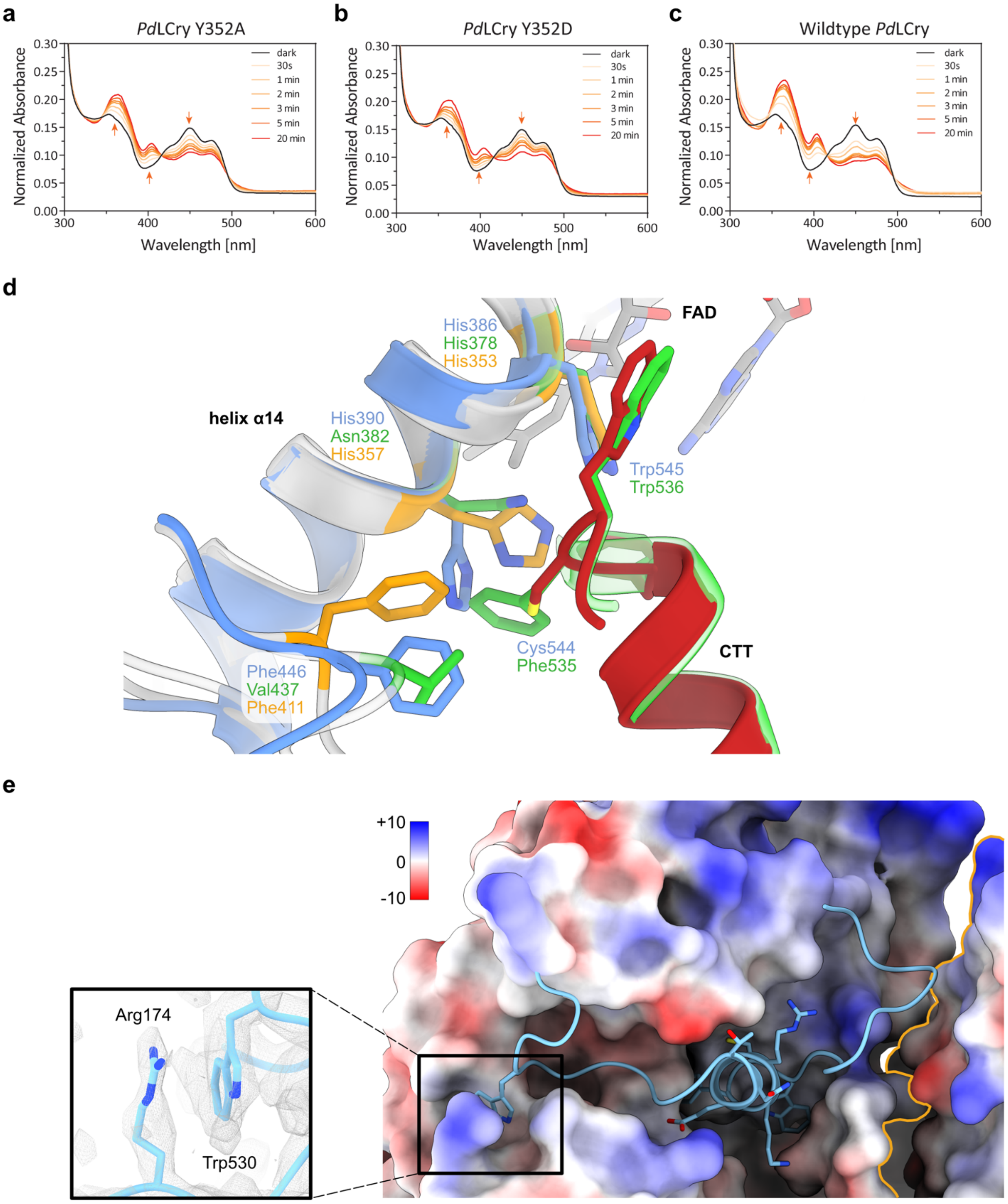

Supplementary Figure 4: Effect of Tyr352 on FAD photoreduction and comparison of CTE binding PHR residues.

a-c Normalized absorption spectra for sunlight photoreduction on ice show that FAD is photoreduced slower and less efficient in the **a** *Pd*LCry^{Y352A} and **b** *Pd*LCry^{Y352D} mutants compared to **c** wildtype *Pd*LCry. Arrows indicate absorbance changes at 370 nm, 404 nm and 450 nm upon photoreduction of dark-state FAD^{OX} (black) to the anionic FAD^{ASQ} radical (dark orange). The spectra show a representative of three independent replicates. **d** Comparison of *Pd*LCry (blue, CTT red), *Dm*Cry (green, PDB 6WTB), and *Cl*Cry4 (orange, PDB 6PU0) interactions with the CTT. Note that the crystallization construct used for solving the structure of *Cl*Cry4 does not contain a CTT, and that our sequence alignment does not show an FFW/FCW-related motif in *Cl*Cry4 (Supplementary Fig. 1). While His386 of *Pd*LCry, which bridges between the CTT and the FAD chromophore, is conserved in all three CRYs, His390 and Phe446 are present only in *Pd*LCry and the type IV CRY *Cl*Cry4. However, *Cl*Cry4 His357 is in a rotamer position reminiscent of *Dm*Cry Asn382, possibly due to the truncation of the CTT in the crystallization construct. *Dm*Cry Phe535 would clash with

His390 of *Pd*LCry. The different position of *Cl*Cry4 Phe411 compared to *Pd*LCry Phe446 could also be due to the absence of a CTT in the truncated crystallization construct. **e** Surface representation of the *Pd*LCry atomic model colored by its electrostatic potential ranging from +10 to -10 kcal/(mol·*e*). The *Pd*LCry CTE (blue ribbon) has an additional anchor N-terminal to the CTT, namely Trp530, which interacts with Arg174 (see insert). The orange line delineates subunit 1 from subunit 2.

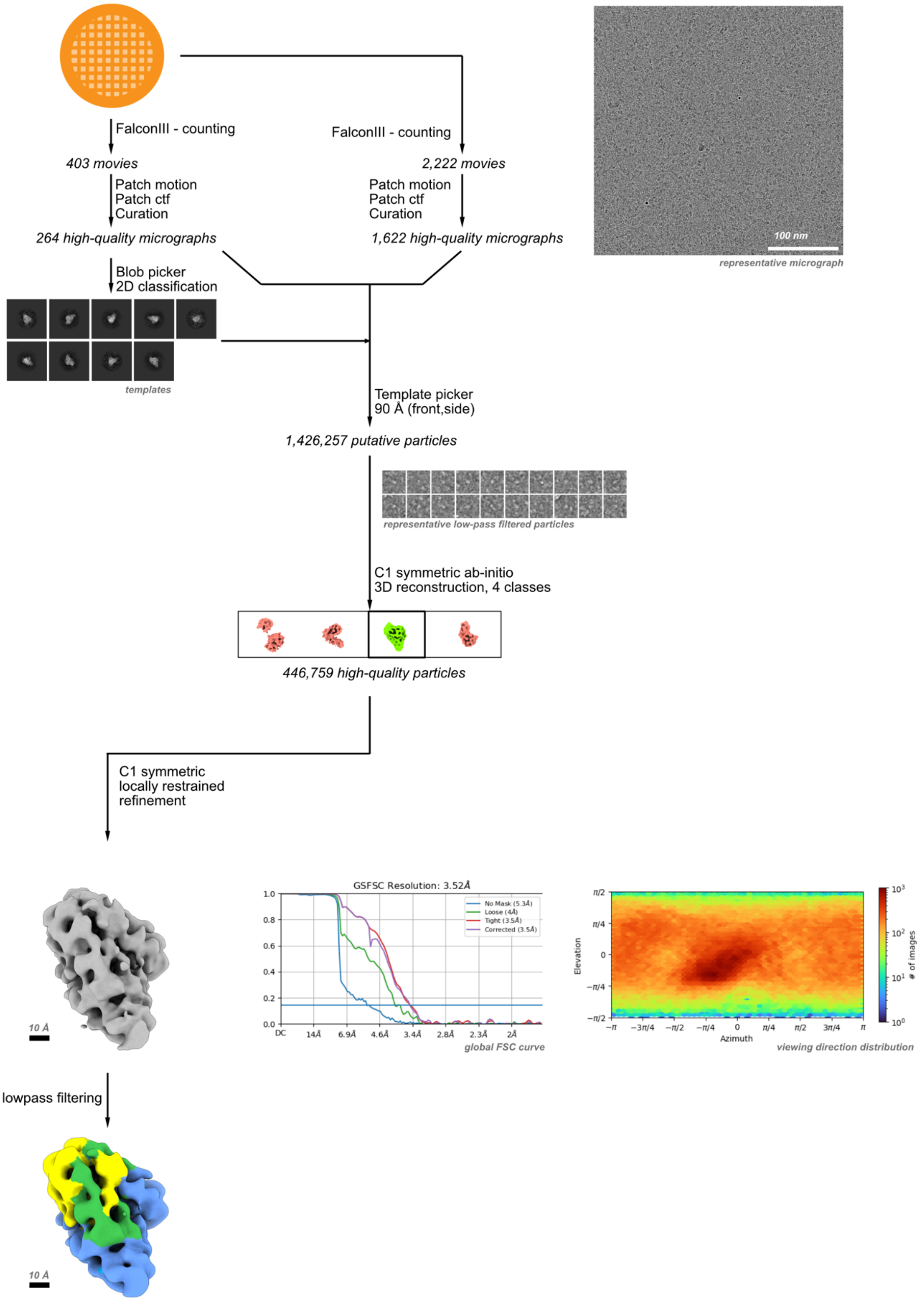

Supplementary Figure 5. Overview of the single particle cryo-EM data processing workflow for the blue light state.

A total of 2 data sets were collected on a 300 kV cryo-electron microscope in the counting mode of the Falcon III detector. Movies were selected for low per-frame drift rates, good CTF scores and low astigmatism. Particles were first picked using a blob picker, and then subjected to unsupervised 2D classification. This resulted, after rejecting clearly artificial classes, in a set of mainly monomeric particles (about 90%), with the remaining classes ambiguous regarding their oligomeric state. Representative classes were used for a template picker, and then curated using unsupervised 2D classification. For further refinement, all particles were classified in 3D using ab-initio reconstruction, sorting into 4

distinct populations. Of these, only one (shown in green and highlighted by a thicker box outline) showed a continuous, protein-like density and particles contributing to this subset were refined in 3D using a non-uniform refinement algorithm with local priors to stabilize the reconstruction, yielding a map with a nominal resolution of 3.5Å, but which was anisotropic. For interpretation we low-pass filtered this map to 8 Å. For further details, see the Materials and Methods section and Supplementary Table 2.

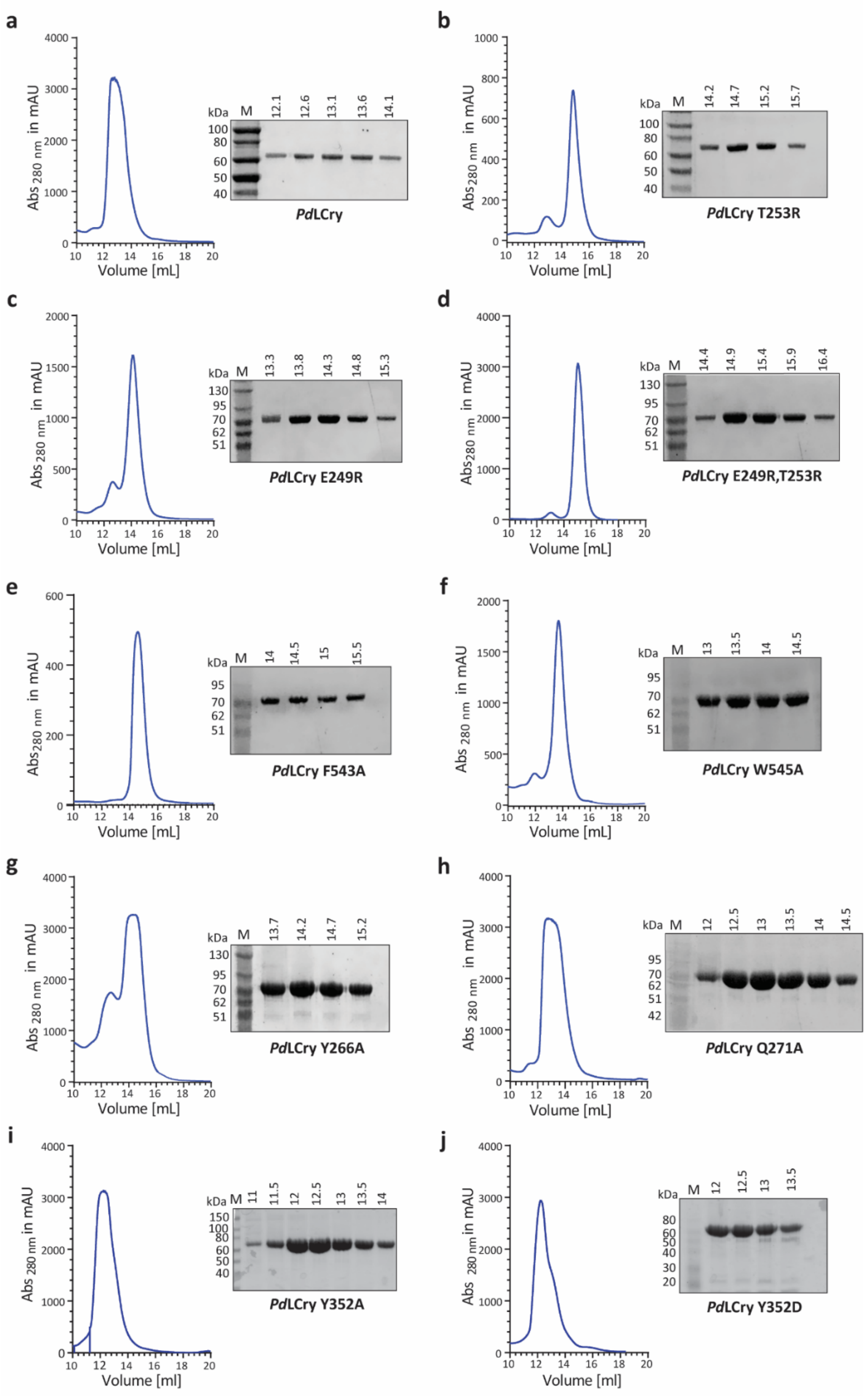

Supplementary Figure 6: Final SEC purification step of wildtype and mutant *Pd*LCry proteins.

Size exclusion chromatography (SEC) chromatograms using an S200 10/300 column (left) and subsequent SDS-PAGE analyses of the SEC peak fractions (right) of **a** wildtype *Pd*LCry, **b** *Pd*LCry$^{\mathrm{T253R}}$, **c** *Pd*LCry$^{\mathrm{E249R}}$, **d** *Pd*LCry$^{\mathrm{E249R,T253R}}$, **e** *Pd*LCry$^{\mathrm{F543A}}$, **f** *Pd*LCry$^{\mathrm{W545A}}$, **g** *Pd*LCry$^{\mathrm{Y266A}}$, **h** *Pd*LCry$^{\mathrm{Q271A}}$, **i** *Pd*LCry$^{\mathrm{Y352A}}$ and **j** *Pd*LCry$^{\mathrm{Y352D}}$. Elution volumes (in mL) of each SEC fraction are indicated on top of the SDS-PAGE gels. M: MW marker. SEC dimer peaks (wt *Pd*LCry, *Pd*LCry$^{\mathrm{Y352A}}$, *Pd*LCry$^{\mathrm{Y352D}}$), total peak (*Pd*LCry$^{\mathrm{Q271A}}$) or monomer peaks (all remaining mutants) were pooled and concentrated for the further analyses.

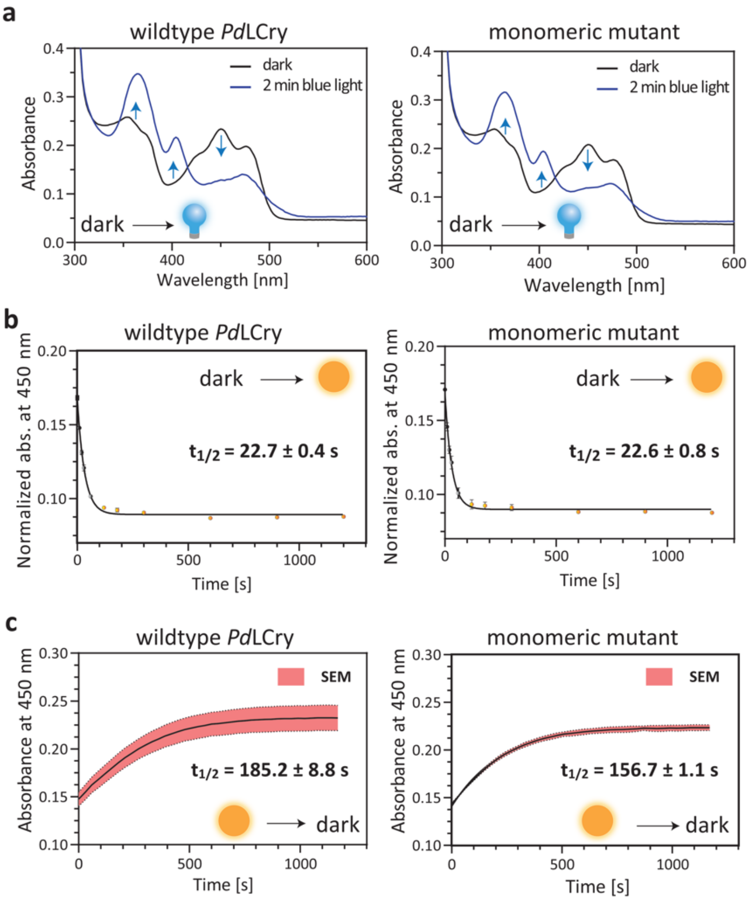

d

	wildtype $t_{1/2}$ [s]	monomer $t_{1/2}$ [s]
Sunlight photoreduction (4°C)	22.1	21.6
	22.7	24.3
	23.4	22.1
Sunlight dark recovery (18°C)	137.1	116.4
	151	122.1
	201	169.6

Supplementary Figure 7: Blue light and sunlight photoreduction and dark recovery kinetics of wildtype *Pd*LCry and the monomeric *Pd*LCry E249R T253R mutant.

a Absorption spectra of wildtype *(left)* and mutant *(right) Pd*LCry in darkness (black) and after 2 min blue light exposure (blue). Arrows indicate the change in absorbance at 370 nm, 404 nm and 450 nm upon photoreduction of FAD^{OX} (black) to the anionic $FAD^{\mathbf{ASQ}}$ radical (blue). **b** Sunlight photoreduction kinetics of wildtype *(left)* and mutant *(right) Pd*LCry following absorbance changes at 450 nm (on ice, ≡ 4°C). Normalized average data points obtained from three independent measurements are plotted. Error bars represent SEM of the three independent replicates. The $t_{1/2}$ values are means of three replicates +/- SEM. **c** Dark recovery kinetics of wildtype *(left)* and mutant *(right) Pd*LCry after sunlight, following absorbance changes at 450 nm at 18°C. Average data points obtained from three independent measurements are plotted. The red shaded region represents the standard error of the mean (SEM) of three independent replicates (at each data point). The $t_{1/2}$ values are means of three replicates +/- SEM. **d** Individual half-live ($t_{1/2}$) values of three independent measurements of sunlight photoreduction and dark recovery kinetics for wildtype *Pd*LCry and the monomeric E249R T253R mutant.

Supplementary Table 1: Sequence and species information

name in figure 1	protein name	species	NCBI/Uniprot ID	comment
AgCry1	cryptochrome 1	*Anopheles gambiae*	ABB29886.1	
AgCry2	cryptochrome 2	*Anopheles gambiae*	ABB29887.1	
AmCry2	cryptochrome 2	*Apis mellifera*	NP_001077099.1	
AquCry1	cryptochrome-2-like	*Amphimedon queenslandica*	XP_003386582.1	
AquCry2	cryptochrome-2-like	*Amphimedon queenslandica*	XP_003386569.1	
AtCry1	cryptochrome 1	*Arabidopsis thaliana*	NP_567341.1	
AtCry2	cryptochrome 2	*Arabidopsis thaliana*	NP_849588.1	
AtCry3	cryptochrome 3	*Arabidopsis thaliana*	NP_568461.3	
ClCry1	cryptochrome 1	*Columba livia*	NP_001302444.1	
ClCry4	cryptochrome 4	*Columba livia*	ANT47196.1	
CmeCry	cryptochrome	*Crateromorpha meyeri*	CAZ66368.1	
CraCry	Uncharacterized protein	*Chlamydomonas reinhardtii*	A8J8W0	from PDB 5ZMO
DmCry	cryptochrome	*Drosophila melanogaster*	NP_732407.1	
DpCry1	cryptochrome 1	*Danaus plexippus*	XP_032522602.1	
DpCry2	cryptochrome 2	*Danaus plexippus*	ABA62409.1	
DrCry3	cryptochrome 3	*Danio rerio*	BAA96850.1	
DrCry4	cryptochrome circadian regulator 4	*Danio rerio*	NP_571862.1	
DrCryDASH	cryptochrome DASH	*Danio rerio*	NP_991249.1	
ErCry1	cryptochrome 1	*Erithacus rubecula*	Q5IZC5.2	
ErCry4	cryptochrome 4	*Erithacus rubecula*	ATE87950.1	
GgCry1	cryptochrome 1	*Gallus gallus*	NP_989576.1	
GgCry2	cryptochrome 2	*Gallus gallus*	NP_989575.1	
GgCry4	cryptochrome 4	*Gallus gallus*	NP_001034685.1	
GviCryDASH	DASH family cryptochrome	*Gloeobacter violaceus*	WP_011140837.1	
HsCry1	cryptochrome 1	*Homo sapiens*	NP_004066.1	
HsCry2	cryptochrome 2	*Homo sapiens*	NP_066940.3	
MmCry1	cryptochrome 1	*Mus musculus*	P97784.1	
MmCry2	cryptochrome 2	*Mus musculus*	Q9R194.1	
PdLCry	light receptive cryptochrome	*Platynereis dumerilii*	UUF95169	
SdoCry	cryptochrome	*Suberites domuncula*	CAZ66367.1	
SeCry	cryptochrome	*Spodoptera exigua*	ADY17887.1	
SlCry1	cryptochrome 1	*Solanum lycopersicum*	NP_001234667.1	
SlCry2	cryptochrome 2	*Solanum lycopersicum*	NP_001234245.1	
SlCryDASH	cryptochrome DASH	*Solanum lycopersicum*	NP_001296304.1	
VcCryDASH	DASH family cryptochrome	*Vibrio cholerae*	WP_000037642.1	
XlCry2	cryptochrome circadian regulator 2 S homeolog	*Xenopus laevis*	NP_001083936.1	
XlCryDASH	cryptochrome DASH	*Xenopus laevis*	NP_001084438.1	
XtCry4	cryptochrome 4 isoform X1	*Xenopus tropicalis*	XP_012812443.1	

Supplementary Table 2: Statistics and additional information of the refinement and model building process

**Dataset dark state**	Cu integration	Au integration	high-dose counting	low-dose counting
**Sample conditions**				
Sample concentration	0.7 mg/ml			
Additive	0.01% fOM			
Grid type	Quantifoil Cu R1.2/1.3. (300 mesh)	UltrAUfoil R1.2/1.3 (300 mesh)		
Light condition	Far-red (>680 nm)			
**Cryo-EM data collection**				
Microscope	Titan Krios G3i			
Voltage (kV)	300			
Spherical aberration Cs (mm)	2.7			
Condenser C2 aperture size (µm)	100	100	50	50
Objective aperture size (µm)	No aperture	No aperture	100	100
Camera	Falcon III			
Pixel size (Å)	0.862			
Total dose (electron*$Å^{-2}$)	66		27	
Number of frames	19		24	48
Exposure time (sec)	0.5		39.5	39.5
Images per hole	2	1	2	2
Energy filter	None			
Defocus range (µm)	-2.0 to -1.0	-1.9 to -1.0	-2.0 to -0.3	-2.0 to -0.3
# micrographs collected	10,368	5,712	1,756	1,679
% micrographs used	72	57	54	47
**Cryo-EM data processing**				
Software	cryoSPARC v3.3.2+220518 patch			
Picked particles	2,697,447	1,910,423	229,982	264,763
Particles after 2D classification	90,729	587,230	69,994	60,987
Particles after 3D sorting	320,005		56,761	47,978
Resolution (FSC 0.143, Å)	2.6			
**Model building and refinement**				
Software for building	Coot 0.9.4.7 EL			
Residues build	31-556			
Software for refinement	PHENIX 1.20.1 - 4487			
Composition (#)<br>Chains<br>Atoms<br>Residues<br>Water<br>Ligands	 2<br>8756 (Hydrogens: 0)<br>Protein: 1052<br>84<br>Mg: 2, FAD: 2			
Bonds (RMSD)<br>Length (Å) (# > 4σ)<br>Angles (°) (# > 4σ)	 0.003<br>0.511			
Ramachandran plot (%)<br>Outliers<br>Allowed<br>Favored	 0<br>3.63<br>96.37			
Rotamer Outliers (%)	0.0			
MolProbity score	1.15			

**Dataset blue light state**	counting
**Sample conditions**	
Sample concentration	0.7 mg/ml
Additive	none
Grid type	UltrAUfoil<br>R1.2/1.3<br>(300 mesh)
Light condition	background illumination: Far-red (>680 nm); sample: 30 sec 455 nm blue LED
**Cryo-EM data collection**	
Microscope	Titan Krios G3i
Voltage (kV)	300
Spherical aberration Cs (mm)	2.7
Condenser C2 aperture size (µm)	70
Objective aperture size (µm)	100
Camera	Falcon III
Pixel size (Å)	0.862
Total dose (electron*Å^{-2})	30
Number of frames	36
Exposure time (sec)	54
Images per hole	1
Energy filter	None
Defocus range (µm)	-2.0 to -0.3
# micrographs collected	2,625
% micrographs used	72
**Cryo-EM data processing**	
Software	cryoSPARC v3.3.2+220518 patch
Picked particles	1,426,257
Particles after 3D sorting	446,759
Resolution (FSC 0.143, Å)	3.5 (filtered to 8 Å)

Supplementary References

1. Czarna, A. *et al.* Structures of Drosophila Cryptochrome and Mouse Cryptochrome1 Provide Insight into Circadian Function. *Cell* **153**, 1394–1405 (2013).
2. Kattnig, D. R., Nielsen, C. & Solov'yov, I. A. Molecular dynamics simulations disclose early stages of the photo-activation of cryptochrome 4. *New J. Phys.* **20**, 083018 (2018).
3. Sjulstok, E., Lüdemann, G., Kubař, T., Elstner, M. & Solov'yov, I. A. Molecular Insights into Variable Electron Transfer in Amphibian Cryptochrome. *Biophys. J.* **114**, 2563–2572 (2018).
4. Solov'yov, I. A., Domratcheva, T., Moughal Shahi, A. R. & Schulten, K. Decrypting Cryptochrome: Revealing the Molecular Identity of the Photoactivation Reaction. *J. Am. Chem. Soc.* **134**, 18046–18052 (2012).
5. Nielsen, C., Nørby, M. S., Kongsted, J. & Solov'yov, I. A. Absorption Spectra of FAD Embedded in Cryptochromes. *J. Phys. Chem. Lett.* **9**, 3618–3623 (2018).
6. Solov'yov, I. A., Domratcheva, T. & Schulten, K. Separation of photo-induced radical pair in cryptochrome to a functionally critical distance. *Sci. Rep.* **4**, 3845 (2014).
