# Supplementary material for: A marine cryptochrome with an inverse photo-oligomerization mechanism
Source: Nat Commun. 2023 Oct 30;14:6918. doi: 10.1038/s41467-023-42708-2 (PMC10616196; doi:10.1038/s41467-023-42708-2)
Supplement: Supplementary file 3 — Reporting Summary [file 41467_2023_42708_MOESM3_ESM.pdf]

nature portfolio

Corresponding author(s): Eva Wolf, Elmar Behrmann

Last updated by author(s): Oct 4, 2023

Reporting Summary

Nature Portfolio wishes to improve the reproducibility of the work that we publish. This form provides structure for consistency and transparency in reporting. For further information on Nature Portfolio policies, see our Editorial Policies and the Editorial Policy Checklist.

Statistics

For all statistical analyses, confirm that the following items are present in the figure legend, table legend, main text, or Methods section.

n/a	Confirmed	
☐	☒	The exact sample size (*n*) for each experimental group/condition, given as a discrete number and unit of measurement
☐	☒	A statement on whether measurements were taken from distinct samples or whether the same sample was measured repeatedly
☒	☐	The statistical test(s) used AND whether they are one- or two-sided<br>*Only common tests should be described solely by name; describe more complex techniques in the Methods section.*
☒	☐	A description of all covariates tested
☒	☐	A description of any assumptions or corrections, such as tests of normality and adjustment for multiple comparisons
☐	☒	A full description of the statistical parameters including central tendency (e.g. means) or other basic estimates (e.g. regression coefficient) AND variation (e.g. standard deviation) or associated estimates of uncertainty (e.g. confidence intervals)
☒	☐	For null hypothesis testing, the test statistic (e.g. *F*, *t*, *r*) with confidence intervals, effect sizes, degrees of freedom and *P* value noted<br>*Give P values as exact values whenever suitable.*
☒	☐	For Bayesian analysis, information on the choice of priors and Markov chain Monte Carlo settings
☒	☐	For hierarchical and complex designs, identification of the appropriate level for tests and full reporting of outcomes
☒	☐	Estimates of effect sizes (e.g. Cohen's *d*, Pearson's *r*), indicating how they were calculated

Our web collection on statistics for biologists contains articles on many of the points above.

Software and code

Policy information about availability of computer code

Data collection	EPU (version 2.12), PurityChrom (version 5.09.115), Spark Control (version 2.2), ChromLab (version 6.1)
Data analysis	MEGA11 (version 11.0.10 build 211109), cryoSPARC (version 3.3.2+220518 patch), Phenix (version 1.20), Coot (version 0.9.4.7), ChimeraX (version 1.5), NAMD (version 2.14), VMD (1.9.3), Jalview (version 2.11), GraphPad PRISM (version 9.5.1-733)

For manuscripts utilizing custom algorithms or software that are central to the research but not yet described in published literature, software must be made available to editors and reviewers. We strongly encourage code deposition in a community repository (e.g. GitHub). See the Nature Portfolio guidelines for submitting code & software for further information.

Data

Policy information about availability of data

All manuscripts must include a data availability statement. This statement should provide the following information, where applicable:

- Accession codes, unique identifiers, or web links for publicly available datasets
- A description of any restrictions on data availability
- For clinical datasets or third party data, please ensure that the statement adheres to our policy

The EM map for the dark state and blue light activated state have been deposited in the EMDB
530 under accession codes EMD-17429 and EMD-17553. Atomic coordinates for dark state PdLCry

531 have been deposited in the Protein Data Bank under the accession code PDB 8P4X. Other data
532 used to generate tables and figures has been provided as source data with this paper.

Research involving human participants, their data, or biological material

Policy information about studies with human participants or human data. See also policy information about sex, gender (identity/presentation), and sexual orientation and race, ethnicity and racism.

Reporting on sex and gender	Our research did not involve human participants, their data or biological material. Sex and gender were not considered in the study design.
Reporting on race, ethnicity, or other socially relevant groupings	Our research did not involve human participants, their data or biological material. Race, ethnicity or other social groupings were not considered in the study design.
Population characteristics	see above
Recruitment	Our research did not involve human participants, their data or biological material.
Ethics oversight	see above

Note that full information on the approval of the study protocol must also be provided in the manuscript.

Field-specific reporting

Please select the one below that is the best fit for your research. If you are not sure, read the appropriate sections before making your selection.

☒ Life sciences ☐ Behavioural & social sciences ☐ Ecological, evolutionary & environmental sciences

For a reference copy of the document with all sections, see nature.com/documents/nr-reporting-summary-flat.pdf

Life sciences study design

All studies must disclose on these points even when the disclosure is negative.

Sample size	Based on the low data spread, three independent samples were determined as sufficient for the UV/Vis experiments
Data exclusions	In general, no data was excluded from the analysis. For the cryo-EM reconstruction, micrographs with poor quality scores (overall motion, CTFfit, astigmatism) were excluded from further analysis - this was however done before evaluating the particle images on them, and thus in a blinded fashion.
Replication	We produced at least three independent replicates of each experiment, using independent samples. Our replicates were consistent, and confirmed reproducibility of the results.
Randomization	Randomization is not relevant for the EM, MD, UV/VIS, SEC and HPLC experiments shown in this study as they are biophysical studies with well-defined parameters and statistics, and their results show low variations that are numerically defined.
Blinding	Due to the design of the freeze-plunger used (transparent door needed for tracking the freezing process), the illumination conditions were not blinded. Similarly, other experiments using light illumination were not blinded. MD simulations could not be blinded as the structures analyzed very visually distinct. A bias due to the omission of blinding is not expected to affect the experiments performed in this study due to their well-defined parameters and statistics, and expected results with low variations that are numerically defined.

Reporting for specific materials, systems and methods

We require information from authors about some types of materials, experimental systems and methods used in many studies. Here, indicate whether each material, system or method listed is relevant to your study. If you are not sure if a list item applies to your research, read the appropriate section before selecting a response.

Materials & experimental systems

n/a	Involved in the study
☒	☐ Antibodies
☐	☒ Eukaryotic cell lines
☒	☐ Palaeontology and archaeology
☒	☐ Animals and other organisms
☒	☐ Clinical data
☒	☐ Dual use research of concern
☒	☐ Plants

Methods

n/a	Involved in the study
☒	☐ ChIP-seq
☒	☐ Flow cytometry
☒	☐ MRI-based neuroimaging

Eukaryotic cell lines

Policy information about cell lines and Sex and Gender in Research

Cell line source(s)	SF9 insect cells (Spodoptera frugiperda, commercial source: Thermo Fisher Scientific. Cat No. 11496015) used for recombinant protein expression (baculovirus infection).
Authentication	Cell lines were not authentificated
Mycoplasma contamination	Not checked for Mycoplasma contamination.
Commonly misidentified lines (See ICLAC register)	no commonly misidentified cell lines were used in the study
